# Integrated Analysis of the Safety Experience in Adults with the Bivalent Respiratory Syncytial Virus Prefusion F Vaccine

**DOI:** 10.3390/vaccines13080827

**Published:** 2025-08-01

**Authors:** Kumar Ilangovan, David Radley, Michael Patton, Emma Shittu, Maria Maddalena Lino, Christos Goulas, Kena A. Swanson, Annaliesa S. Anderson, Alejandra Gurtman, Iona Munjal

**Affiliations:** 1 Vaccine Research & Development, Pfizer Inc., Pearl River, NY 10965, USA; 2 Vaccine Research & Development, Pfizer Ltd., Marlow SL7 1YL, UK; 3 Worldwide Safety, Pfizer Srl, 20152 Milan, Italy; 4 Worldwide Safety, Pfizer Hellas SA, 55535 Thessaloniki, Greece

**Keywords:** clinical trial, RSV, RSVpreF, safety, vaccine

## Abstract

**Background/objectives:** This was a post hoc analysis of safety data across the bivalent respiratory syncytial virus prefusion F (RSVpreF) vaccine clinical trial development program. **Methods:** Data from eight clinical trials in 46,913 immunocompetent adults who received RSVpreF or placebo were analyzed. Local reactions and systemic events were assessed among non-pregnant ≥18-year-olds (*n* = 9517); adverse events (AEs) among pregnant and non-pregnant 18–59-year-olds (*n* = 9238); and vaccine-related AEs among non-pregnant ≥18-year-olds (*n* = 39,314). Post-marketing data in non-pregnant adults were considered. **Results:** Local reactions and systemic events were reported more frequently in RSVpreF versus placebo recipients; injection site pain was the most common local reaction (RSVpreF, 18.9%; placebo, 7.4%), and fatigue (23.5%; 18.4%) and headache (19.5%; 15.0%) were the most common systemic events. Percentages of AEs within 1 month after vaccination were similar across groups (RSVpreF, 12.8%; placebo, 13.1%); severe AEs were reported in ≤1.5% of participants. Differences in percentages of individuals reporting vaccine-related AEs between the RSVpreF and placebo groups were <0.2% for all related AEs. Serious AEs throughout the study were reported in ≤14.0% (RSVpreF, 12.6%; placebo, 14.0%). No atrial fibrillation, Guillain-Barré syndrome, or acute polyneuropathy cases were reported. The AE data from post-marketing data sources were consistent with the safety profile from the clinical trial program, with no new safety concerns. **Conclusions:** Integrated data demonstrated that RSVpreF was well tolerated with a favorable safety profile in non-pregnant and pregnant adults. Ongoing surveillance through real-world use and clinical trial experience continue to support the safety profile of RSVpreF. ClinicalTrials.gov: NCT03529773/NCT04071158/NCT04785612/NCT05035212/NCT05096208/NCT05842967/NCT04032093/NCT04424316.

## 1. Introduction

Respiratory syncytial virus (RSV)-associated illnesses affect individuals across all age groups, inducing a range of symptoms from mild cold-like illness to severe disease manifestations of lower respiratory tract illness and exacerbation of underlying chronic cardiopulmonary and immunocompromising conditions [1,2,3]. Severe RSV disease can lead to hospitalization and RSV-related death [4,5]. Although infants and older adults are among those at highest risk of severe RSV-associated illness, there remains substantial RSV disease burden among adult populations 18 through 59 years of age that is not only limited to individuals with underlying comorbidities [4,5,6,7]. RSV is estimated to affect approximately 64 million people globally each year, with up to 10,000 deaths in individuals 65 years of age and older [8]. A 2020 meta-analysis reported that RSV was responsible for between 1% and 10% of influenza-like acute respiratory tract illness among individuals 50 years of age or older worldwide [9]. In the United States, RSV illness is associated with 110,000 to 180,000 hospitalizations each year among individuals 50 years of age and older [10]. However the true burden of RSV disease is likely to be underestimated [11,12].

Prophylaxis against RSV-associated illness through vaccination is available for certain populations. RSVpreF (Abrysvo^®^; Pfizer Inc, New York, NY, USA) is a bivalent RSV-A/B prefusion F protein vaccine that received initial US approval in 2023 based predominantly on phase 3 clinical efficacy study results [13,14,15]. RSVpreF is administered at the 120 µg dose level and is currently licensed in the United States for the prevention of RSV-associated lower respiratory tract illness in adults ≥60 years of age and those 18 through 59 years of age at high risk of severe RSV-associated disease, as well as in infants via maternal vaccination [13]. In the European Union, RSVpreF is additionally licensed in all adults 18 years and older [16]. Two other vaccines are available for prevention of RSV-associated lower respiratory tract illness in adults ≥60 years of age and adults at high risk of severe RSV illness: an RSV-A prefusion F protein vaccine (Arexvy^®^; GlaxoSmithKline, Durham, NC, USA) and an mRNA-based RSV-A vaccine encoding the prefusion F protein (mRESVIA^®^; Moderna, Princetown, NJ, USA) [17,18,19].

RSVpreF has been evaluated in several clinical studies in adult immunocompetent populations [14,15,20,21,22,23,24,25,26,27]. In each of these clinical trials, RSVpreF had an acceptable safety and tolerability profile. While demonstrating vaccine safety is a crucial consideration for licensure and implementation of vaccination programs, individual clinical trials are typically insufficiently powered to identify potential rare or very rare safety events [28]. Therefore, pooled analyses of safety data are an invaluable approach for identifying rare safety events and supporting the safety and tolerability profile of a vaccine. Here we report a large, pooled analysis of clinical studies and post-marketing experience that assessed the safety and reactogenicity of RSVpreF among individuals ≥18 years of age.

## 2. Methods

### 2.1. Design

This was a post hoc integrated analysis of reactogenicity and safety data from the RSVpreF (Abrysvo^®^) clinical trial program and post-marketing experience among healthy adults. Eight clinical trials were included in this analysis, specifically 5 clinical trials in male and non-pregnant female participants 18 through 59 years of age (a first-in-human trial; a lot consistency trial; a human challenge trial; a noninferiority trial with co-administered tetanus, diphtheria, and acellular pertussis vaccine; and a trial in participants at high risk of severe RSV illness), 2 clinical trials in pregnant female participants 18 through 49 years of age, and 1 clinical trial in male and female participants ≥60 years of age (Table 1) [14,15,20,21,22,23,24,25,26,27]. Real-world post-marketing data of spontaneous adverse event (AE) reports in non-pregnant adults were also included. All clinical trial protocols for each trial in this combined analysis were evaluated by an ethics committee before the start of each study, as has been reported previously [14,15,20,21,22,23,24,25,26,27].

### 2.2. Safety Parameters

To support the reactogenicity profile of RSVpreF among adults, local reactions (i.e., injection site pain, redness, and swelling) and systemic events (i.e., fever, fatigue, headache, muscle pain, diarrhea, joint pain, nausea, and vomiting) occurring within 7 days after vaccination among the clinical trial population of male and non-pregnant female participants ≥18 years of age were assessed. Severe reactogenicity events were classified as those preventing daily activity or fever (>38.9 °C), vomiting (requiring intravenous hydration), and diarrhea (≥6 loose stools in 24 h). Local reactions and systemic events by demographic subgroup, including age (18–59 years; ≥60 years), sex, race (White; Black; Asian), and ethnicity (Hispanic/Latino; non-Hispanic/non-Latino), were determined.

Adverse drug reactions (ADRs), for which there was a reason to conclude that RSVpreF caused the event(s) [29], were categorized as very common (≥1/10), common (≥1/100 to <1/10), or rare (≥1/10,000 to <1/1000). ADRs are adverse effects in which the association between the medicinal product and the adverse effect is likely and is typically based on late-stage analyses [30]. 

To support the AE profile for RSVpreF 120 µg (the licensed dose [13,31]) among individuals 18 through 59 years of age, data from healthy participants within this age group, including both male and pregnant and non-pregnant female participants from the clinical trial program who received either unadjuvanted RSVpreF 120 µg or placebo, were pooled in the safety database; this provided a comprehensive summary of safety in the general healthy population of 18- through 59-year-olds. Safety parameters included AEs reported through 1 month after vaccination and serious AEs (SAEs) collected throughout the studies. Reports of AEs of special interest (AESIs; atrial fibrillation, Guillain-Barré syndrome [GBS], and polyneuropathy) were collected throughout the study. AEs from the clinical trial program and spontaneous post-marketing reports from the Pfizer global safety database were coded by the Medical Dictionary for Regulatory Activities system organ class and preferred terms. Analysis of post-marketing reports was performed using a data lock point of 30 November 2024.

### 2.3. Statistical Analysis

Descriptive statistics were calculated, including percentages of participants experiencing AEs and 95% CIs determined by the Clopper and Pearson method. This was selected over asymptotic methods to avoid lower bounds below zero since many AEs have low incidence rates. For differences in percentages of participants with related AEs, exact 2-sided 95% CIs were calculated using the Miettinen and Nurminen method, which has been shown to have accurate coverage probability [32]. Post-marketing data are presented descriptively.

## 3. Results

### 3.1. Populations

**Figure 1:**
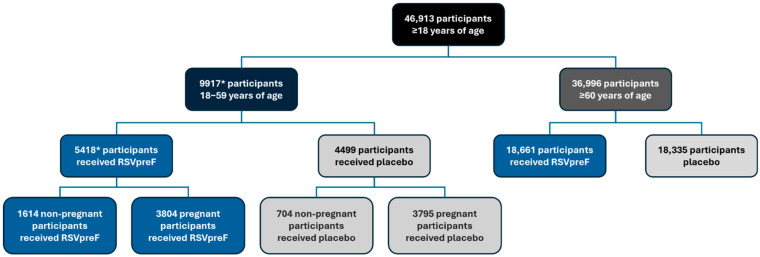
Pooled clinical trial safety population. RSVpreF recipients received RSVpreF 120 µg without adjuvant either alone or concomitantly with placebo, SIIV, or Tdap. Placebo recipients received placebo alone or with SIIV or Tdap. See Table 1 for further details of the clinical trials included. * Includes 1 participant from the RENOIR trial in adults ≥60 years of age who was 59 years of age at the time of study vaccination. RSVpreF, bivalent RSV prefusion F vaccine; SIIV, seasonal inactivated influenza vaccine; Tdap, tetanus, diphtheria, and acellular pertussis vaccine.

The pooled safety database included 46,913 participants 18 years and older (Figure 1). This population included 9917 participants 18 through 59 years of age (RSVpreF, *n* = 5418; placebo, *n* = 4499) and 36,996 participants ≥60 years of age (RSVpreF, *n* = 18,661; placebo, *n* = 18,335). Among the participants 18 through 59 years of age, 7599 were pregnant (RSVpreF, *n* = 3804; placebo, *n* = 3795).

Among the population of participants 18 through 59 years of age, 92.2% (9147/9916) were female, 66.3% (6578/9916) were White, 19.9% (1973/9916) were Black, 10.3% (1025/9916) were Asian, and 72.1% (7150/9916) were non-Hispanic/non-Latino (Table S1). Most participants in this population were 18 through 49 years of age (96.6% [9580/9916]), and 58.3% (5778/9916) were from the United States, 9.8% (973/9916) from South Africa, and 9.5% (938/9916) from Argentina.

### 3.2. Local Reactions and Systemic Events Among ≥18-Year-Olds

**Figure 2:**
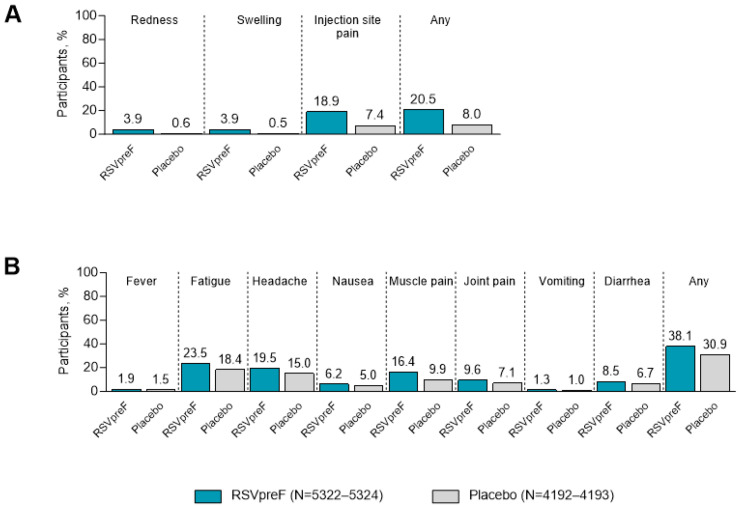
Local reactions (**A**) and systemic events (**B**) among non-pregnant adults ≥18 years of age. Includes pooled data from the following studies: C3671001, C3671004, WI257521, a subset of RENOIR, C3671014, and MONET. RSVpreF recipients received RSVpreF 120 µg without adjuvant either alone or concomitantly with placebo, SIIV, or Tdap. RSVpreF, bivalent RSV prefusion F vaccine; SIIV, seasonal inactivated influenza vaccine; Tdap, tetanus, diphtheria, and acellular pertussis vaccine.

In this pooled analysis, data for local reactions and systemic events are based on 9517 non-pregnant individuals ≥18 years of age, including a subset of participants ≥60 years of age (i.e., 7209 participants in this age group who completed electronic diaries). Local reactions were reported more frequently overall in participants who received RSVpreF (20.5% [1092/5324]) compared with placebo (8.0% [336/4193]; Figure 2A); similarly, systemic events were reported more frequently in RSVpreF recipients (38.1% [2030/5324]) compared with placebo recipients (30.9% [1296/4193]; Figure 2B). The most frequently reported local reaction was injection site pain (RSVpreF, 18.9% [1005/5322]; placebo 7.4% [309/4192]), and the most frequently reported systemic events were fatigue (RSVpreF, 23.5% [1250/5324]; placebo 18.4% [771/4193]) and headache (RSVpreF, 19.5% [1040/5324]; placebo 15.0% [627/4193]). Most reactogenicity events were mild to moderate in severity; severe local reactions were reported in 0.3% (14/5324) of RSVpreF recipients (vs. <0.1% [2/4193] of placebo recipients), and severe systemic events were reported in 1.3% (68/5324) and 0.7% (31/4193) of RSVpreF and placebo recipients, respectively.

When analyzed by demographic subgroups, local reactions and systemic events were generally more commonly reported among 18- through 59-year-old versus ≥60-year-old participants and among female versus male participants (Figures S1 and S2). Local reactions and systemic events were reported by similar percentages of participants when analyzed by race, but individuals of non-Hispanic/non-Latino ethnicity reported more reactogenicity events than those of Hispanic/Latino ethnicity.

### 3.3. Adverse Drug Reactions Among ≥18-Year-Olds

Among the pooled population of 5324 non-pregnant RSVpreF recipients ≥18 years of age, very common ADRs were identified as fatigue, headache, injection site pain, and muscle pain. Common ADRs included vaccination site redness, vaccination site swelling, and arthralgia. Rare ADRs included rash (generally related to the injection site) and lymphadenopathy.

### 3.4. Adverse Events Among 18- Through 59-Year-Olds

**Figure 3:**
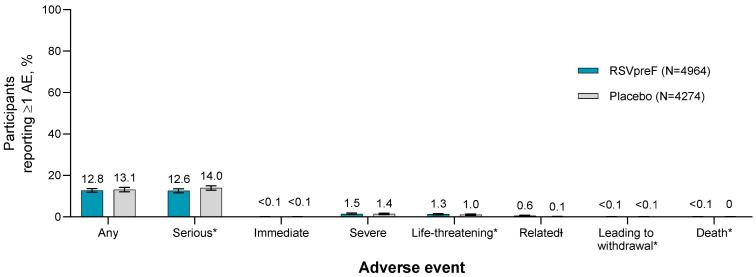
Summary of AEs within 1 month after vaccination or throughout the study * among adults 18 through 59 years of age. * Adverse events are reported through 1 month after vaccination; serious and life-threatening AEs, AEs leading to withdrawal, and deaths are reported throughout the study period. Ɨ Related AEs were assessed by the investigator as being related to the vaccine or placebo. Includes pooled data from the following studies: C3671001, C3671003, C3671004, C3671014, MATISSE, WI257521. Values above the bars are percentages of participants reporting ≥1 event. Two-sided 95% CIs were calculated using the Clopper–Pearson method. AE, adverse event; RSVpreF, bivalent RSV prefusion F vaccine.

Among the pooled population of 9238 male and non-pregnant and pregnant female participants 18 through 59 years of age, the percentages reporting any AE within 1 month after vaccination were similar in the RSVpreF group (12.8% [634/4964]) and the placebo group (13.1% [562/4274]; Figure 3). Severe AEs were reported in ≤1.5% of participants across both groups (RSVpreF, 1.5% [76/4964]; placebo, 1.4% [59/4274]). AEs assessed as related to the study intervention by the investigator were more frequent in the RSVpreF group (0.6% [28/4964]) compared with the placebo group (0.1% [6/4274]). Immediate AEs (within 30 min of vaccination) and AEs leading to withdrawal throughout the studies were reported by <0.1% of participants across study groups. Throughout the studies, SAEs were reported in ≤14.0% (RSVpreF, 12.6% [623/4964]; placebo, 14.0% [597/4274]) of participants, including potentially life-threatening AEs in ≤1.3% (RSVpreF, 1.3% [64/4964]; placebo, 1.0% [44/4274]) of participants. Two deaths were reported, neither of which was considered related to study vaccination: 1 postpartum hemorrhage and hypovolvemic shock during delivery 57 days after vaccination in a maternal participant who received RSVpreF in the MATISSE study and 1 toxicity to various agents 181 days after vaccination (i.e., quetiapine and amlodipine) in a non-pregnant female participant who received RSVpreF in study C3671001. A detailed review by the investigator and the sponsor of both fatal AEs reaffirmed a lack of causality.

**Figure 4:**
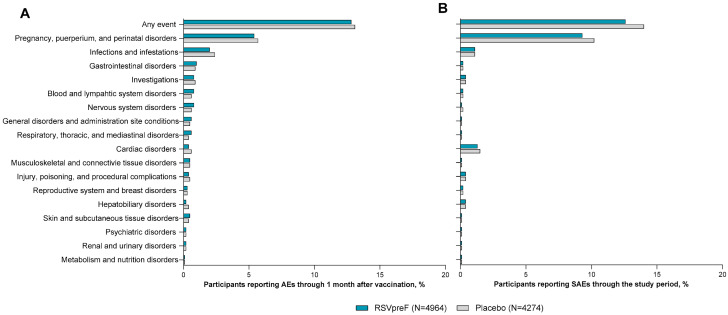
AEs reported within 1 month of vaccination (**A**) and serious AEs reported from vaccination throughout the study period (**B**) by system organ class in adults 18 through 59 years of age. Includes pooled data from the following studies: C3671001, C3671003, C3671004, C3671014, MATISSE, and WI257521. Data are shown for system organ classes in which ≥0.1% of participants reported an AE overall. AE, adverse event; RSVpreF, bivalent RSV prefusion F vaccine; SAE, serious adverse event.

**Figure 5:**
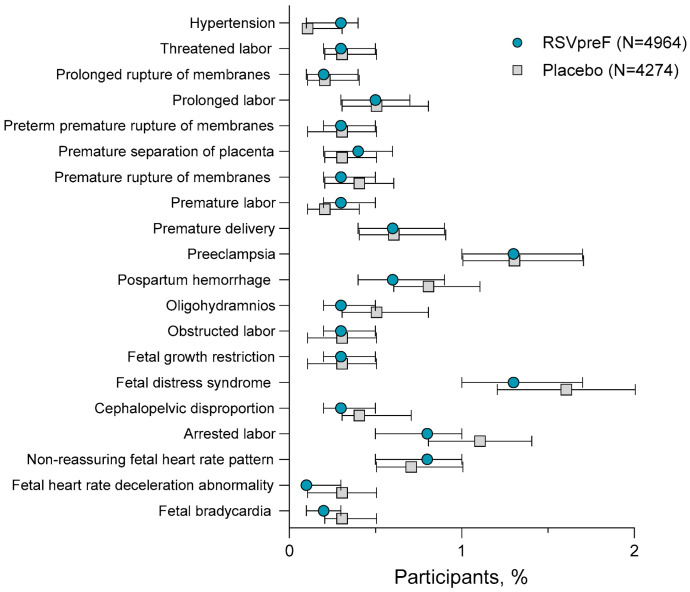
Most commonly reported SAEs * from vaccination throughout the study period in adults 18 through 59 years of age. * SAEs reported in ≥0.2% of participants. Includes pooled data from the following studies: C3671001, C3671003, C3671004, C3671014, MATISSE, and WI257521. Two-sided 95% CIs were calculated using the Clopper–Pearson method. AE, adverse event; RSVpreF, bivalent RSV prefusion F vaccine; SAE, serious adverse event.

The most commonly reported AEs within 1 month of vaccination by system organ class were pregnancy, puerperium, and perinatal conditions (RSVpreF, 5.7% [269/4964]); placebo, 5.7% [245/4274]) and infections and infestations (RSVpreF, 2.0% [100/4964]; placebo, 2.4% [103/4274]; Figure 4A). Most of these events were considered unrelated to study vaccine. Pregnancy, puerperium, and perinatal conditions (RSVpreF, 9.3% [460/4964]; placebo, 10.2% [436/4274]), cardiac disorders (1.3% [63/4964]; 1.5% [66/4274]), and infections and infestations (1.1% [55/4964]; 1.1% [47/4274]) were the most common SAEs by system organ class reported throughout the studies (Figure 4B). The most commonly reported SAE by preferred terms throughout the study period were preeclampsia (RSVpreF, 1.3% [67/4964]; placebo, 1.3% [56/4274]) and fetal distress syndrome (RSVpreF, 1.3% [67/4964]; placebo, 1.6% [67/4274]; Figure 5). SAEs were reported at similar frequencies in RSVpreF and placebo groups, with overlapping 2-sided 95% CIs. No cases of atrial fibrillation, GBS, or acute polyneuropathy without an underlying etiology (i.e., AESIs) were reported in the pooled population of 9238 male and non-pregnant and pregnant female participants 18 through 59 years of age.

### 3.5. Related Adverse Events Among ≥18-Year-Olds

**Figure 6:**
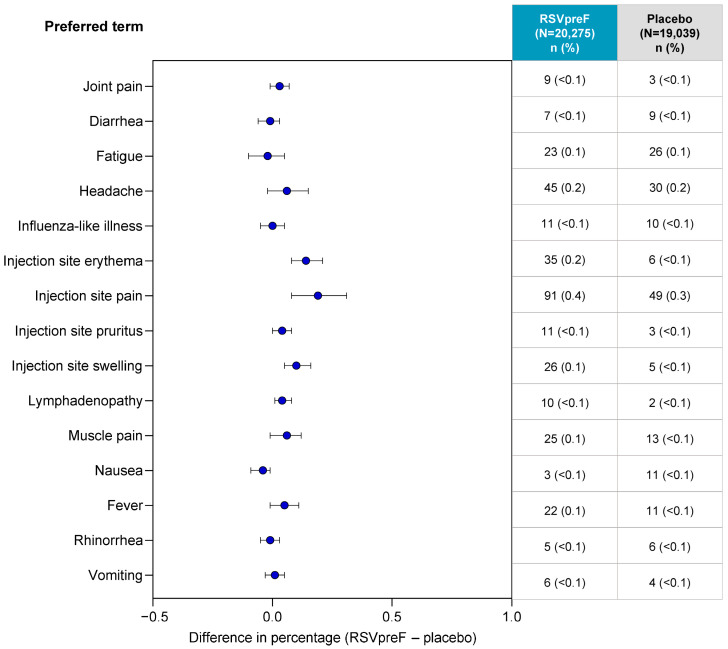
Comparison of related AEs * within 1 month after vaccination. * AEs considered to be related to vaccination and reported in ≥10 participants (≥0.025%) overall. Includes pooled data from the following studies: C3671001, C3671004, WI257521, RENOIR, C3671014, and MONET. Any AE reported was included, regardless if it was considered as reactogenicity. RSVpreF recipients received RSVpreF 120 µg without adjuvant either alone or concomitantly with placebo, SIIV, or Tdap. Placebo recipients received placebo alone or with SIIV or Tdap. The difference was calculated as the percentage of participants with AEs for RSVpreF 120-μg group minus the percentage of participants in the placebo group; 2-sided 95% CIs were based on the Miettinen and Nurminen method. The table shows the number and percentage of participants reporting. Preferred terms were coded from MedDRA (v26.1). AE, adverse event; RSVpreF, bivalent RSV prefusion F vaccine; SIIV, seasonal inactivated influenza vaccine; Tdap, tetanus, diphtheria, and acellular pertussis vaccine.

Among the pooled population of 39,314 non-pregnant individuals ≥18 years of age, the difference in percentage of individuals reporting vaccine-related AEs between the RSVpreF and placebo groups through 1 month after vaccination was minimal (<0.2% for all related AEs; Figure 6). Related AEs with the largest difference between RSVpreF and placebo groups were injection site pain (difference, 0.19%; RSVpreF, 0.4% [91/20,275]; placebo, 0.3% [49/19,039]) and injection site erythema (difference, 0.14%; RSVpreF, 0.2% [35/20,275]; placebo, <0.1% [6/19,039]).

### 3.6. Post-Marketing Adverse Events

As of 30 November 2024, nearly 20 million doses of RSVpreF had been shipped globally. At this time among non-pregnant adults ≥18 years of age, 3519 cumulative AEs had been reported from post-marketing data sources in 1252 individuals. Most cases originated in the United States (50.2%) and the United Kingdom (42.2%). The most common AEs by system organ class in the reporting period were general disorders and administration site conditions (21.9% of AEs [771/3519]; Figure S3A). The most common AEs by preferred term were headache and fatigue (3.2% [112/3519] and 2.8% [99/3519] of cases, respectively; Figure S3B). The majority of reports were in older adults (59.7% [748/1252] in participants ≥65 years of age), a minority were in younger adults (10.4% [130/1252] in participants 18–64 years of age), and age was not provided in 29.9% (374/1252) of individuals.

GBS was reported in 49 post-marketing AEs, which were individually reviewed. After review, 40 of these 49 cases had either insufficient evidence to meet or did not meet Brighton Collaboration levels of evidence 1–3 (i.e., diagnostic certainty) for GBS [33]. Among all 49 reports, 2 cases reported a latency not suggestive of a temporal relationship with vaccination (symptom onset same day as vaccination) and 5 cases were preceded by an infection. No RSVpreF vaccine batch number was reported for nearly 50% of the cases, and the reported batch number was not compatible with the Pfizer batch number for an additional 8 cases. Most cases had multiple underlying comorbidities; at least 10 reports listed a medical history that could represent a predisposing factor for GBS or an alternative etiology to GBS (including multiple sclerosis, underlying polyneuropathy, and cancer). Among the 9 reports that met the diagnostic certainty for GBS, 3 included a preceding infection that suggested a possible alternative etiology. Multiple comorbidities were reported in the medical history of all 9 cases, including autoimmune diseases, cardiac issues, peripheral neuropathy (1 case) and previous GBS (1 case). In 5 cases, the RSV vaccine was co-administered with other vaccines (herpes zoster, influenza, rabies, COVID-19, and pneumococcal vaccines). Overall, most of the reported cases included insufficient elements to perform a final causality assessment.

## 4. Discussion

This pooled analysis of the RSVpreF clinical study program and post-marketing experience shows that RSVpreF has an acceptable safety profile in adults. Although there is a high risk of poor outcomes from RSV-associated disease in immunocompromised individuals [34,35], there is also an unmet need to prevent RSV in immunocompetent young adults, a population that was the focus of this pooled analysis. For non–high-risk adult populations 18 through 59 years of age, management of RSV disease is generally limited to supportive measures (hydration, oxygenation, and limited use of aerosolized ribavirin) [36,37,38]. Natural infection with RSV also does not generate long-term immunity, with recurrent infections occurring for both adults and children [39]. Reports of RSV disease incidence among adults vary across global populations and is frequently underestimated within the published literature for multiple reasons, including RSV testing infrequency, differences in RSV case definitions, time frame of reported incidence, and use of a single diagnostic specimen in most settings [5,40,41,42,43].

The burden of RSV-associated disease among adults is difficult to ascertain because of limited surveillance systems, particularly outside of high-income regions [12]. A global meta-analysis of high-income countries estimated 537,000 RSV-related hospitalizations among adults <65 years of age with RSV-related hospitalization case fatality rates of 5.7% (range, 4.7%–7.0%) compared with 6.1% (range, 3.3%–11.0%) for adults ≥65 years of age [44,45]. A systematic literature review and meta-analysis describing population-based rates of medically attended RSV among US adults reported a substantial annual burden of medically attended RSV disease for those <65 years of age, with more than 3 million outpatient visits, approximately 350,000 emergency department visits, and about 60,000 hospitalizations [4]. Vaccination against RSV in adults <60 years of age could therefore help prevent a substantial disease burden. 

In this pooled safety and tolerability analysis, the most commonly reported reactogenicity events were injection site pain, fatigue, and headache; these events were generally mild or moderate and are commonly seen after vaccinations in adults, including with COVID-19, influenza, hepatitis, meningococcal, and other RSV vaccines [46,47,48,49,50]. In subgroup analyses, both local reactions and systemic events tended to be reported more frequently in participants <60 years of age and females compared with those ≥60 years of age and males. A number of intrinsic factors can affect the reactogenicity profile of a vaccine, including age, sex, ethnicity, and body mass index [51]. The phenomena of increased reactogenicity among younger adults or those of female sex have been reported previously for other vaccines, including COVID-19 and influenza vaccines [52,53,54]. The mechanisms behind the lower reactogenicity in the older compared with younger participants receiving RSVpreF are not fully understood but are not thought to be due to any decrease in protective neutralizing immune response [15]. A potential explanation for increased reactogenicity among individuals of female sex is due to manifestation of higher innate immune responses compared with individuals of male sex [55,56,57]. Differences between sexes in adaptive immunity, genetics, hormones, and the microbiome may also be contributary [57].This pooled analysis of reactogenicity did not include pregnant individuals; however, reactogenicity during pregnancy has been previously reported and is consistent with these analyses [14].

The percentage of non-pregnant and pregnant participants reporting any AEs within 1 month after vaccination was similar in the RSVpreF and placebo group, with >98.5% of AEs being mild or moderate in severity. AEs through 1 month and SAEs throughout the study were most commonly reported in the pregnancy, puerperium, and perinatal conditions system organ class at similar frequencies between RSVpreF and placebo groups. The US prescribing information notes preeclampsia and gestational hypertension as maternal adverse reactions and states that RSVpreF should be administered to pregnant individuals at 32 through 36 weeks gestational age to avoid potential risk of preterm birth [13]; AEs during pregnancy have been well described elsewhere [14,27]. AEs that were considered to be related to vaccination occurring at greater frequencies in the RSVpreF group versus placebo were most commonly reactogenicity-type events, although these differences were reassuringly minimal and consistent with the favorable reactogenicity profile from a Centers for Disease Control and Prevention review of the benefits and harms of protein subunit RSV vaccines [58].

Vaccine-associated GBS has been reported after respiratory virus infections and at a much lower frequency with several vaccines, including COVID-19, herpes zoster, and influenza vaccines, particularly among older adults [59,60,61,62]. Two cases of GBS variants with confounding etiologies were also reported in the phase 3 RENOIR efficacy trial in adults ≥60 years of age, of whom 18,574 participants received RSVpreF [15]. In the current analysis, no cases of GBS were reported in the pooled clinical trial data of participants 18 through 59 years of age. GBS diagnosis met Brighton Collaboration criteria levels of evidence 1–3 (diagnostic certainty) in 9 post-marketing reports after distribution of nearly 20 million doses of RSVpreF; nevertheless, a definitive causality assessment cannot be performed due to incomplete information reported. As with all vaccines, continued surveillance of potential vaccine-associated GBS after RSVpreF remains important.

The review of global post-marketing data, based on reports largely originating from the United States and the United Kingdom, showed a safety profile consistent with that observed in clinical studies with no new safety concerns. Post-marketing surveillance of vaccines is critical to increase the possibility of detecting rare events that may not be observed in clinical trials [63,64,65]; the absence of new safety signals after distribution of almost 20 million doses of RSVpreF globally is therefore reassuring. Limitations of post-marketing reports include the voluntary nature of submissions and that the extent of underreporting is not known. There are several factors that may influence whether an event is reported, including the length of time since marketing of the drug or vaccine, market share of the drug or vaccine, publicity about a drug or vaccine, publicity about an AE, the seriousness of the event, regulatory actions, healthcare provider and consumer awareness of adverse drug event reporting, and litigation. Given that many external factors influence AE reporting, the spontaneous reporting system yields reporting proportions rather than incidence rates. Therefore, between-drug or vaccine comparisons using these proportions are generally not appropriate; rather, the spontaneous reporting system should be used for signal detection and not hypothesis testing. Clinical information (including medical history, time from drug or vaccine use to illness onset, dose, validation of diagnosis, and concomitant drug use) may be missing or incomplete, and there may be no or limited follow-up. Accrual of post-marketing AE reports therefore does not indicate definitively that a particular event was caused by the drug or vaccine; rather, the event may be due to underlying disease or other factor, including medical history or concomitant medication.

A key strength of this analysis was pooling of safety data across rigorously conducted controlled clinical trials from a large overall population size, increasing the possibility that rare and very rare AEs will be detected. The descriptive nature of the statistical analysis is consistent with pooled safety analyses of other vaccines [66,67,68,69,70]. Limitations of the analysis include that different populations were used for different analyses. Although adults 18 through 59 years of age were the focus of this analysis, adults ≥60 years of age were included in some of the analyses, such as reactogenicity and related AEs; inclusion of older adults in the reactogenicity analyses allowed for age subgroup comparisons, while their inclusion in the related AE analysis allowed for better comparison with placebo due to the larger sample size. Finally, because it is not possible to obtain an accurate estimate of RSVpreF doses administered from post-marketing sources, the true prevalence of post-marketing AE reports cannot be determined.

## 5. Conclusions

In conclusion, safety data from 8 clinical trials and post-marketing surveillance demonstrated that RSVpreF was well tolerated in non-pregnant and pregnant individuals ≥18 years of age. Continued post-marketing safety monitoring of RSVpreF administered during routine clinical practice and ongoing follow-up from clinical trials will provide further data.

## Figures and Tables

**Table 1:** Summary of RSVpreF clinical trials included in the safety analyses.

Study Name/Number (NCT Number)	Design	Location	Population	Number of Participants ≥18 Years of Age	
				Total Randomized	Included in Safety Analysis
C3671001 (NCT03529773) [20,21]	Phase 1/2 randomized, placebo-controlled, observer-blind, dose-finding first-in-human study	United States	Healthy male and non-pregnant female participants 50–85 years of age	617	291 *
C3671004 (NCT04071158) [22]	Phase 2b randomized, placebo-controlled, multi-center, observer-blind, noninferiority study	United States	Healthy non-pregnant female participants 18–49 years of age receiving concomitant Tdap	427	421
WI257521 (NCT04785612) [23]	Phase 2a, randomized, placebo-controlled, single-center, double-blind, exploratory human challenge study	United Kingdom	Healthy male and non-pregnant female participants 19–50 years of age	70	70
RENOIR (NCT05035212) [15]	Phase 3, randomized, placebo-controlled, multi-center, double-blind study	Argentina, Canada, Finland, Japan, Netherlands, South Africa, United States	Healthy ^†^ male and non-pregnant participants 59–97 years of age	36,862	36,862
C3671014 (NCT05096208) [24]	Phase 3, randomized, placebo-controlled, multi-center, double-blind, lot consistency study	United States	Healthy male and non-pregnant female participants 18–49 years of age	993	992
MONET Substudy A (NCT05842967) [25]	Phase 3, randomized, placebo-controlled, multi-center, double-blind study	United States	Healthy male and non-pregnant female participants 18–59 years of age and at high risk of severe RSV disease	681	678
C3671003 (NCT04032093) [26]	Phase 2b, observer-blinded, randomized, placebo-controlled, dose-finding and proof-of-concept study	Argentina, Chile, South Africa, United States	Healthy pregnant female participants 18–49 years of age	232	232
MATISSE (NCT04424316) [14,27]	Phase 3, multi-center, double-blinded, placebo-controlled trial	Argentina, Australia, Brazil, Canada, Chile, Denmark, Finland, Gambia, Japan, Mexico, Netherlands, New Zealand, Philippines, Republic of Korea, South Africa, Spain, Taiwan, United States	Healthy pregnant female participants 14–47 years of age	7420	7367

RSVpreF, bivalent RSV prefusion F vaccine; Tdap, tetanus, diphtheria, and acellular pertussis vaccine. * Includes 156 participants 50 through 59 years of age; Dose 1 only. ^†^ Or with stable medical conditions.

## Data Availability

Upon request, and subject to review, Pfizer will provide the data that support the findings of this study. Subject to certain criteria, conditions, and exceptions, Pfizer may also provide access to the related individual de-identified participant data. See https://www.pfizer.com/science/clinical-trials/trial-data-and-results (accessed on 29 July 2025) for more information.

## Supplementary Materials

The following supporting information can be downloaded at: https://www.mdpi.com/article/10.3390/vaccines13080827/s1, Figure S1. Local reactions by subgroup. Figure S2. Systemic events by subgroup. Figure S3. Number of most common* AEs of any cause reported during post-marketing surveillance of RSVpreF by system organ class (A) and preferred term (B). Table S1. Demographic and baseline characteristics among participants 18 through 59 years of age.
