# Supplementary material for: Integrated Analysis of the Safety Experience in Adults with the Bivalent Respiratory Syncytial Virus Prefusion F Vaccine
Source: Vaccines (Basel). 2025 Aug 1;13(8):827. doi: 10.3390/vaccines13080827 (PMC12390091; doi:10.3390/vaccines13080827)

Figure S1. Local reactions by subgroup

Includes pooled data from the following studies: C3671001, C3671004, WI257521, a subset of RENOIR, C3671014, and MONET. RSVpreF, bivalent RSV prefusion F vaccine.

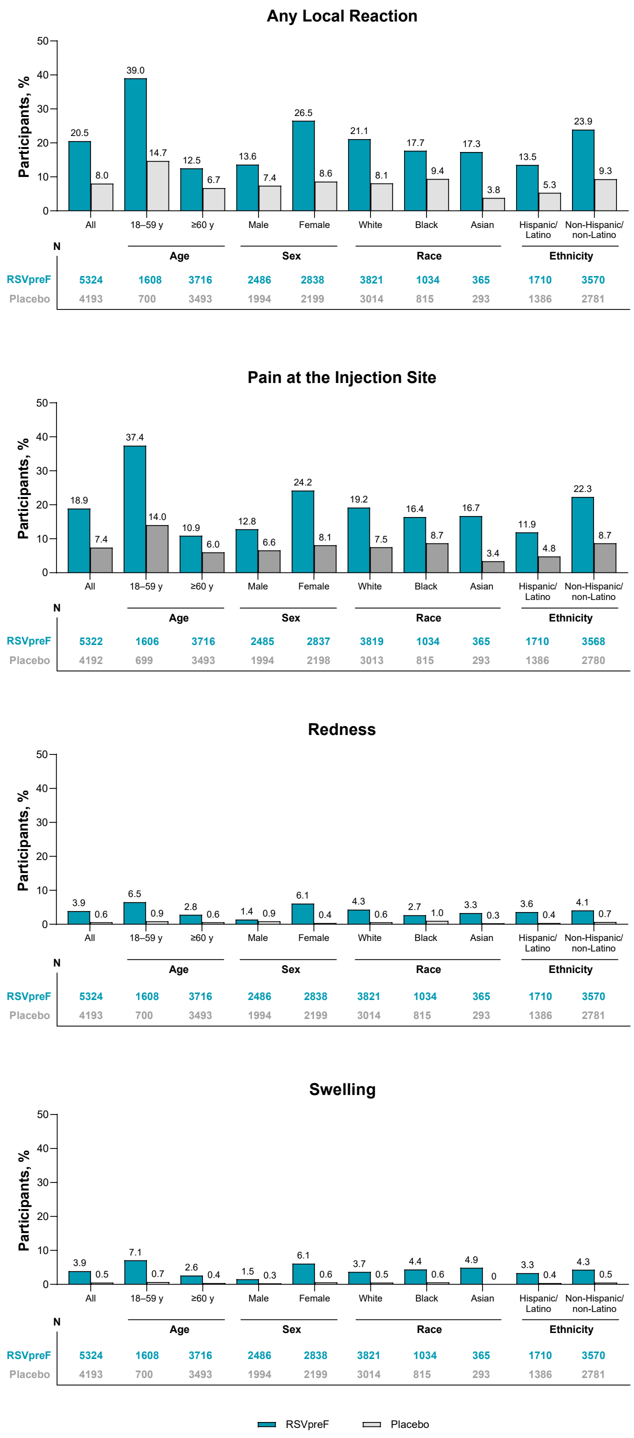

Supplement: Supplementary file 1 [file vaccines-13-00827-s001.zip › vaccines-3703649_Figure S1.pdf]
