# Supplementary material for: Integrated Analysis of the Safety Experience in Adults with the Bivalent Respiratory Syncytial Virus Prefusion F Vaccine
Source: Vaccines (Basel). 2025 Aug 1;13(8):827. doi: 10.3390/vaccines13080827 (PMC12390091; doi:10.3390/vaccines13080827)
Supplement: Supplementary file 1 [file vaccines-13-00827-s001.zip › vaccines-3703649_Figure S2.pdf]

Figure S2. Systemic events by subgroup

Includes pooled data from the following studies: C3671001, C3671004, WI257521, a subset of RENOIR, C3671014, and MONET. RSVpreF, bivalent RSV prefusion F vaccine

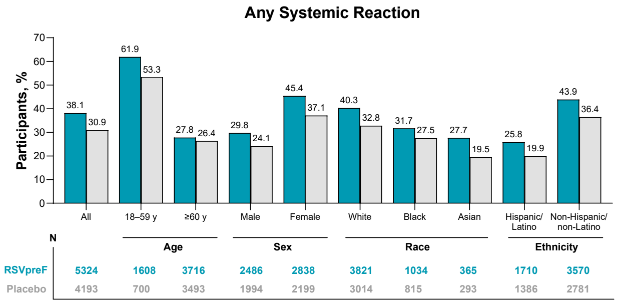

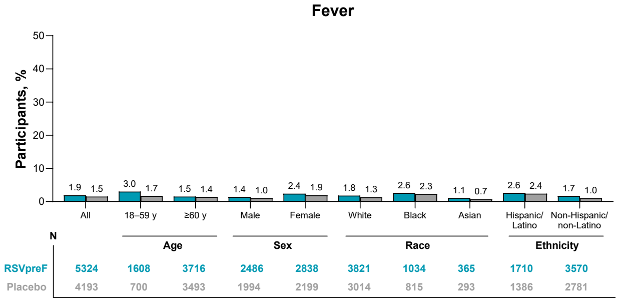

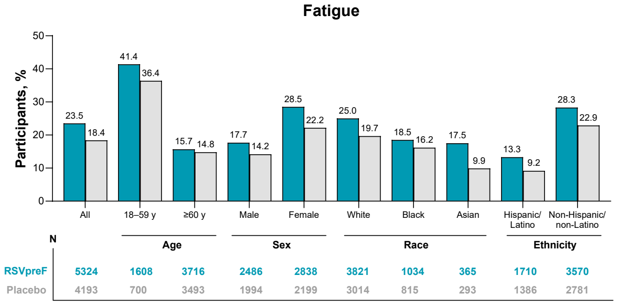

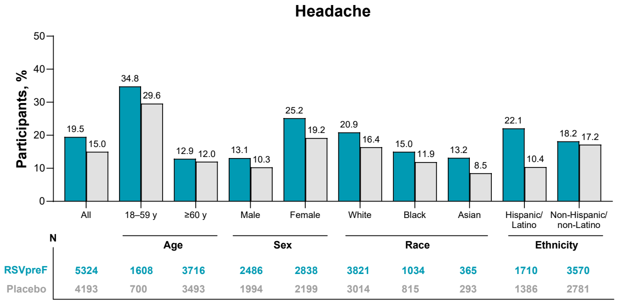

Nausea

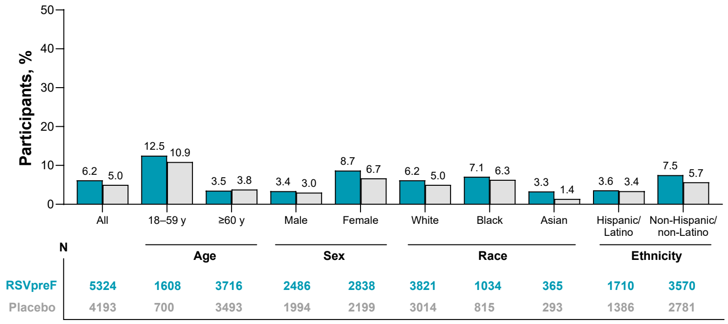

Muscle Pain

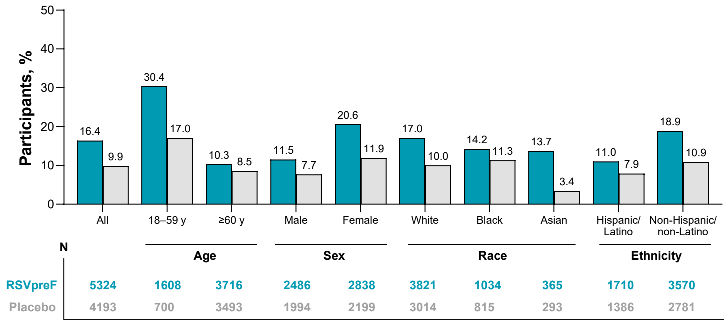

Joint Pain

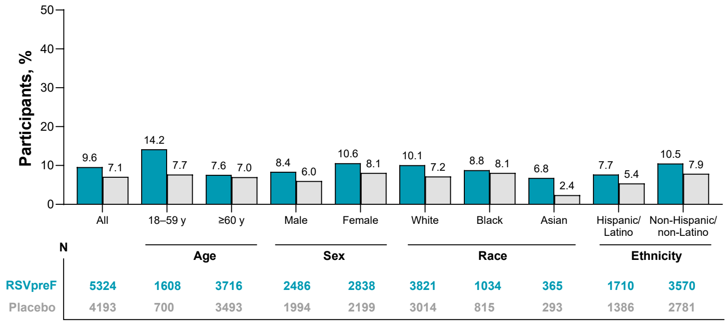

Vomiting

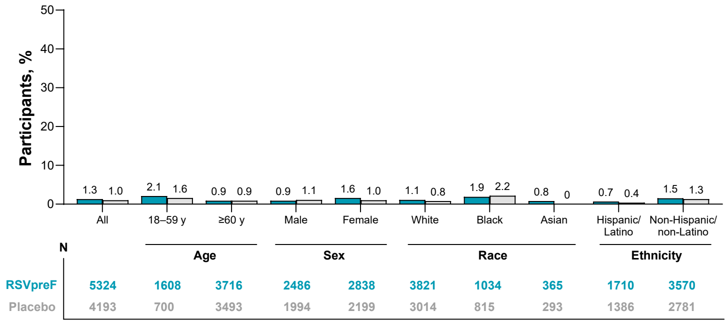

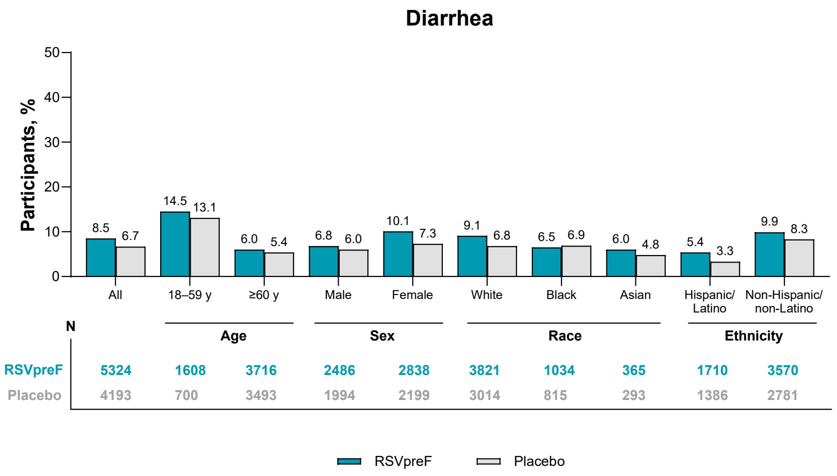
Diarrhea
Participants, %
0
10
20
30
40
50
8.5
6.7
14.5
13.1
6.0
5.4
6.8
6.0
10.1
7.3
9.1
6.8
6.5
6.9
6.0
4.8
5.4
3.3
9.9
8.3
All
18–59 y
≥60 y
Male
Female
White
Black
Asian
Hispanic/ Latino
Non-Hispanic/ non-Latino
Age
Sex
Race
Ethnicity
N
RSVpreF 5324 1608 3716 2486 2838 3821 1034 365 1710 3570
Placebo 4193 700 3493 1994 2199 3014 815 293 1386 2781
RSVpreF
Placebo
