# Supplementary material for: Integrated Analysis of the Safety Experience in Adults with the Bivalent Respiratory Syncytial Virus Prefusion F Vaccine
Source: Vaccines (Basel). 2025 Aug 1;13(8):827. doi: 10.3390/vaccines13080827 (PMC12390091; doi:10.3390/vaccines13080827)

Figure S3. Number of most common* AEs of any cause reported during post-marketing surveillance of RSVpreF by system organ class (A) and preferred term (B)

* In Panel A, data include AEs reported at a frequency of >25; in Panel B, data include AEs reported at a frequency of >50. Non-serious and serious AEs were designated as reported. AE, adverse event; RSVpreF, bivalent RSV prefusion F vaccine.

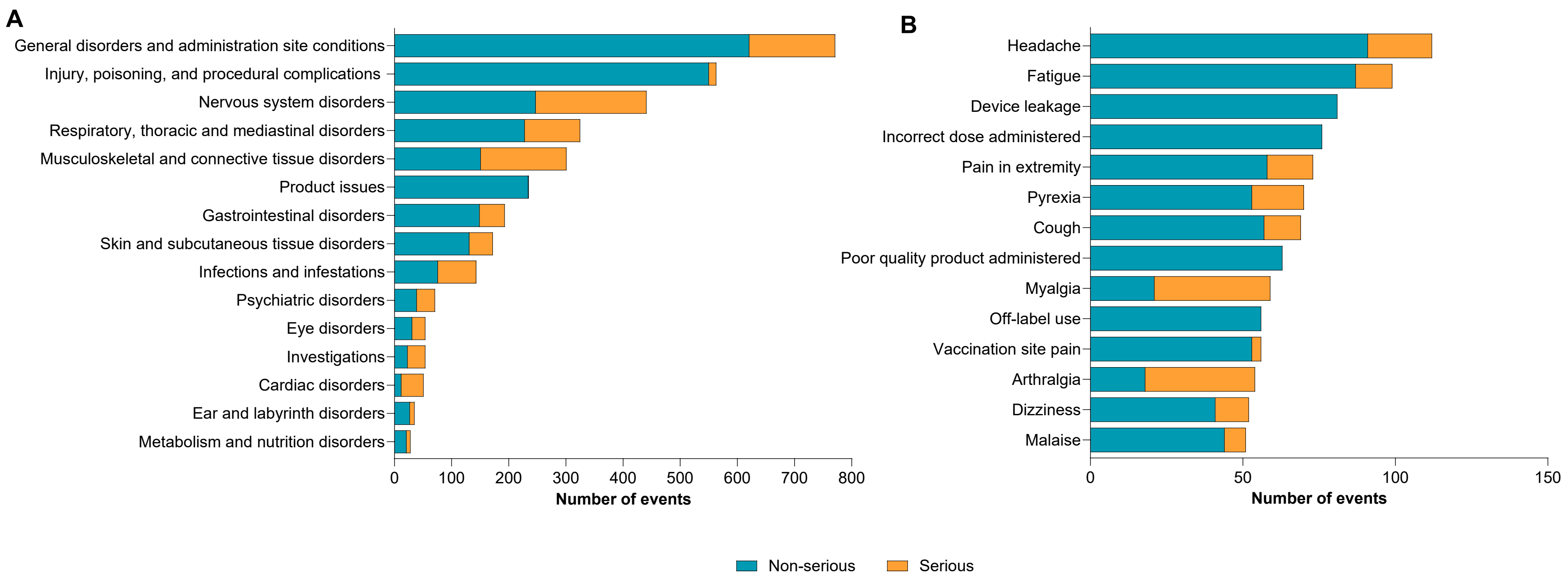

Supplement: Supplementary file 1 [file vaccines-13-00827-s001.zip › vaccines-3703649_Figure S3.pdf]
