# Supplementary material for: Integrated Analysis of the Safety Experience in Adults with the Bivalent Respiratory Syncytial Virus Prefusion F Vaccine
Source: Vaccines (Basel). 2025 Aug 1;13(8):827. doi: 10.3390/vaccines13080827 (PMC12390091; doi:10.3390/vaccines13080827)
Supplement: Supplementary file 1 [file vaccines-13-00827-s001.zip › vaccines-3703649_Table S1.pdf]

Table S1. Demographic and baseline characteristics among participants 18 through 59 years of age

Characteristic	RSVpreF (N=5417*)	Control (N=4499)	Total (N=9916)
Sex, *n* (%)			
Male	542 (10.0)	227 (5.0)	769 (7.8)
Female	4875 (90.0)	4272 (95.0)	9147 (92.2)
Race, *n* (%)			
White	3616 (66.8)	2962 (65.8)	6578 (66.3)
Black	1078 (19.9)	895 (19.9)	1973 (19.9)
Asian	536 (9.9)	489 (10.9)	1025 (10.3)
American Indian or Alaskan Native	48 (0.9)	42 (0.9)	90 (0.9)
Native Hawaiian or other Pacific Islander	17 (0.3)	15 (0.3)	32 (0.3)
Multi-racial	49 (0.9)	33 (0.7)	82 (0.8)
Not reported	60 (1.1)	54 (1.2)	114 (1.1)
Other or unknown	13 (0.2)	9 (0.2)	22 (0.2)
Ethnicity, *n* (%)			
Hispanic/Latino	1435 (26.5)	1244 (27.7)	2679 (27.0)
Non-Hispanic/non-Latino	3933 (72.6)	3217 (71.5)	7150 (72.1)
Other or unknown	49 (0.9)	38 (0.8)	87 (0.9)
Age at vaccination, years			
18–49, *n* (%)	5200 (96.0)	4380 (97.4)	9580 (96.6)
50–59, *n* (%)	217 (4.0)	119 (2.6)	336 (3.4)
Country, *n* (%)			
United States	3345 (61.8)	2433 (54.1)	5778 (58.3)
South Africa	487 (9.0)	486 (10.8)	973 (9.8)
Argentina	471 (8.7)	467 (10.4)	938 (9.5)
Japan	232 (4.3)	232 (5.2)	464 (4.7)
Taiwan	130 (2.4)	130 (2.9)	260 (2.6)
Spain	117 (2.2)	122 (2.7)	239 (2.4)
Gambia	98 (1.8)	98 (2.2)	196 (2.0)
Netherlands	97 (1.8)	95 (2.1)	192 (1.9)
Chile	90 (1.7)	89 (2.0)	179 (1.8)
Finland	75 (1.4)	73 (1.6)	148 (1.5)
New Zealand	50 (0.9)	49 (1.1)	99 (1.0)
Philippines	40 (0.7)	39 (0.9)	79 (0.8)
Mexico	38 (0.7)	37 (0.8)	75 (0.8)
Brazil	36 (0.7)	37 (0.8)	73 (0.7)

United Kingdom	35 (0.6)	35 (0.8)	70 (0.7)
Denmark	31 (0.6)	31 (0.7)	62 (0.6)
Canada	27 (0.5)	28 (0.6)	55 (0.6)
Australia	11 (0.2)	13 (0.3)	24 (0.2)
South Korea	7 (0.1)	5 (0.1)	12 (0.1)

RSVpreF, bivalent RSV prefusion F vaccine.
Includes pooled data from the following studies: C3671001, C3671003, C3671004, WI257521, C3671014, MATISSE, and MONET.
*1 participant 59 years of age was enrolled in the RENOIR trial in participants ≥60 years of age, and included in the integrated analysis of safety but is not included in this demographics table.
